# False Memory in Alzheimer's Disease

**DOI:** 10.1155/2020/5284504

**Published:** 2020-02-19

**Authors:** Mohamad El Haj, Fabienne Colombel, Dimitrios Kapogiannis, Karim Gallouj

**Affiliations:** ^1^Univ Nantes, Laboratoire de Psychologie des Pays de la Loire (LPPL), EA 4638, F-44000 Nantes, France; ^2^Unité de Gériatrie, Centre Hospitalier de Tourcoing, Tourcoing, France; ^3^Institut Universitaire de France, Paris, France; ^4^Laboratory of Neurosciences, National Institute on Aging, Baltimore, MD, USA

## Abstract

Patients with Alzheimer's Disease (AD) not only are suffering from amnesia but also are prone to memory distortions, such as experiencing detailed and vivid recollections of episodic events that have never been encountered (i.e., false memories). To describe and explain these distortions, we offer a review to synthesize current knowledge on false memory in AD into a framework allowing for better understanding of the taxonomy and phenomenology of false memories and of the cognitive mechanisms that may underlie false memory formation in AD. According to this review, certain phenomenological characteristics of memories (e.g., high emotional load, high vividness, or high familiarity) result in misattributions in AD. More specifically, this review proposes that generalized decline in cognitive control and inhibition in AD may result in difficulties in suppressing irrelevant information during memory monitoring, especially when irrelevant (i.e., false) information is characterized by high emotion, vividness, or familiarity. This review also proposes that binding deficits in AD decrease the ability to retrieve relevant contextual details, leading to source monitoring errors and false memories. In short, this review depicts how phenomenological characteristics of memories and failures of monitoring during retrieval contribute to the occurrence of false memory in AD.

## 1. Introduction

Consider the following scenario, you are driving on a busy road when you perceive someone waiting at a bus stop. You may believe that you have already met this person and wonder where, when, and how you met her. You may also wonder whether you met this person at one of the weekly meetings at your company. At the next crossroad, you may realize that you have never met this person before. This illustration shows how you may create a false memory about a stimulus (in our example, a person waiting at a bus stop) or even about an entire episode (in our example, that you met that person at that meeting). While in everyday life we are occasionally prone to form innocuous false memories (e.g., we may falsely believe in response to someone smiling at us in the street, “I probably met this person before”), these false attributions are more frequent and less innocuous in patients with Alzheimer's Disease (AD). Critically, as we will show below, false memory in AD does involve not only a stimulus (e.g., a smiling face at the street) but also an entire episode; for instance, a patient may falsely remember that a person is a close friend or may misattribute information from news stories and commercials [1] or even information related to a national traumatic event, such as the attacks of September 11, 2001 [2].

False memories in AD may be associated with serious consequences as the patients may act upon their false beliefs. For instance, a patient may give money to a person acting on the false memory that that person is a close friend. A patient may also falsely remember hiding money somewhere and accuse family members/caregivers of stealing it. Further illustrating the consequences of false memory, a patient may submit a payment based on the false memory that she ordered something, or, conversely, a patient may refuse to pay for an item based on the false memory that she has already done so. A daily concern of caregivers of AD patients is that a patient may skip a medication dose acting on the false memory that she has already taken it.

Motivated by the clinical importance of false memories in AD, and recognizing the limits of the current theoretical framework accounting for these memories, we offer a comprehensive overview of the cognitive characteristics of these memories in AD. More precisely, we propose a hypothesis to describe how phenomenological characteristics (e.g., emotion, vividness, and familiarity) of false memories may influence memory monitoring (i.e., attribution regarding the origin of memories). This review also proposes a set of hypotheses about the domains of executive function that may be involved in false memories. Also, this review demonstrates how false memory in AD involves perceptions (e.g., a face), spatiotemporal context (“where/when/how I met that person?”), and entire autobiographical events (e.g., “that person is a neighbor and we used to drink tea together”). Note that this review distinguishes between false context memory and false autobiographical memory, in that false context memory concerns distortions of the ability to remember the context in which an information was previously encoded (i.e., where/when/how the information was encoded), whereas false autobiographical memory concerns distortions of memories of personal events. While this dissociation follows naturally from theoretical models that emphasize context memory [3] or autobiographical memory [4, 5], research on false memory in AD has been concerned either with context memory or with autobiographical memory. This divergence can be explained by the tendency of research on false context memory in AD that utilizes experimental paradigms with a limited number of controlled variables that are administered in the laboratory, whereas research on false autobiographical memory mainly deals with clinical situations and examines memory within the ecological context of patients' everyday life. The laboratory-centric approach to false context memory in AD has been also adopted by researchers studying false item memory in the disease. Notably, autobiographical memory has been neglected by research assessing false item memory in AD.

To summarize, research on false memory in AD has investigated item memory, context memory, and, to a lesser degree, autobiographical memory separately, and scarce, if any, theoretical attempts have been undertaken to gather and systematize available knowledge or classify the different forms of false memory in AD. Moreover, research on false memories in AD has rarely been concerned with their phenomenological characteristics, an important parameter because it may allow us to understand how AD patients may misattribute their memories. As we will discuss, AD patients may misattribute memories with high emotional load, vividness, or familiarity. To address these issues, we propose below a classification of the different forms of false memories in AD. We also describe how phenomenological characteristics of memories may influence misattributions in AD.

## 2. False Item Memory, False Context Memory, and False Autobiographical Memory

### 2.1. False Item Memory

The range of false memories that may be encountered in AD includes false item memory, false context memory, and false autobiographical memory. To begin with false item memory, research on it has typically used the Deese–Roediger–McDermott paradigm [6, 7]. In this paradigm, participants are typically presented with lists of words thematically linked (e.g., travel, wagon, and locomotive) and all semantically associated with a single nonpresented word called a critical lure (e.g., train). Words are generally presented in a descending order of associative strength, with the most strongly associated items being presented first. After presentation of a list, participants are then subjected to a free recall task (immediate or deferred) and/or a recognition task. In healthy participants, research typically demonstrates a strong tendency to recall and recognize the critical lure falsely. The production of the critical lure has been attributed by the activation-monitoring theory [8, 9] to a propagation process from trace activation to encoding, associated with failure of the monitoring process responsible for identifying the source of acquired information, at the time of its recuperation. Research using the Deese–Roediger–McDermott paradigm has demonstrated a decrease in correct responses and an increase in false memories with normal aging [10, 11] which has been attributed to failures in source monitoring [12]. Regarding AD, research using the Deese–Roediger–McDermott paradigm has demonstrated an increase in false recalls or false recognition of critical lures in AD patients [13]. Recently, Evrard, Gilet, Colombel, Dufermont, and Corson [14] presented AD patients with two lists, one with descending forward associative strength and one with ascending forward associative strength. In the first list, words most strongly associated with a critical lure were at the beginning, and those least strongly associated with a critical lure were presented at the end. In the second list, the order of presentation of the words was reversed; those least strongly associated with a critical lure were at the beginning, and those most strongly associated with it were presented at the end. The authors observed that when words were presented in a forward associative strength order, AD patients produced no more critical lures than healthy older adults, whereas when words were presented in the ascending forward associative strength order, AD patients produced more critical lures compared to healthy older participants. The authors suggested that, when the strongest associates of the critical lure were placed at the end of the list, the critical lure produced high levels of memory activation in AD patients. Consequently, the feeling of familiarity for the critical lure increased leading to a reduction of the ability of AD patients to control the source of the activation, leading to the high production of the critical lure for the ascending forward associative strength list. These findings suggest how the order of presentation of information may provoke false item memory in AD patients.

False item memory in AD, as observed in research using the Deese–Roediger–McDermott paradigm, can also be interpreted in light of the Fuzzy Trace Theory [15]. This theory proposes that memory is based on the encoding and retrieval of two different types of representations: a global (i.e., gist) representation of the meaning of the event and a much more detailed representation with a focus on the specificity of events. According to this theory, the erroneous recall of the critical lure in the Deese–Roediger–McDermott paradigm is the result of a gist processing of information rather than a detailed one. Supporting this assumption, Gallo et al. [8] attributed production of critical lures in AD to an overdependence on gist memory. This overdependence can be attributed to disruption in semantic memory and/or to attention deficit that may prevent AD patients from accessing the general theme of the list and therefore the critical lure [16]. Another explanation is that AD patients activate the critical lure in semantic memory, but, due to episodic memory deficits, memory traces associated with the lure (e.g., how/when the lure was previously presented) vanish quickly [17]. False item memory in AD, as observed in research using the Deese–Roediger–McDermott paradigm, can also be understood in light of a study by Gilet et al. [13] who reported lower production of critical lures in AD patients than controls. The authors suggested that memory traces of the critical lure, normally preactivated during encoding, subsequently become unavailable, making it difficult for patients to recall the critical lure. In summary, false item memory in AD, as typically reported by research using the Deese–Roediger–McDermott paradigm, can be attributed to several factors, such as overdependence on gist memory during encoding, difficulties to maintain memory traces during encoding, and/or deficits of source monitoring during retrieval.

### 2.2. False Context Memory

Unlike research on item memory, research on false context memory has been mainly interested in the ability of AD patients to monitor the context in which an item was previously encoded. Research on context memory has demonstrated significant levels of false alarms in AD patients, i.e., incorrect statements by patients that a novel context had been encountered previously. In this body of research, context memory was evaluated regarding different contextual dimensions (e.g., where, when, and how an information was previously encoded). For instance, Goldman, Winograd, Goldstein, O'Jile, and Green [18] invited AD patients to decide whether certain facts were previously told by the experimenter or by someone else. In another study [19], AD patients were required to decide whether objects were previously manipulated by themselves or by the experimenter. The difficulties of AD patients to monitor context memory were also evaluated by Mammarella, Fairfield, and Di Domenico [20] who invited AD patients to retain different contextual attributes of words (perceptual, spatial, temporal, semantic, social, and affective details of words). Results demonstrated false recognition for the contextual attributes of these words in AD patients. A similar conclusion was reached by Pierce, Waring, Schacter, and Budson [21] who reported difficulties of AD patients in remembering in what room items were previously encoded.

The ability to monitor the encoding context was also evaluated with respect to the ability of AD patients to distinguish between enacted and imagined events. Mammarella et al. [20] reported difficulties in AD patients to remember whether they previously performed or imagined performing actions such as folding a paper. A tendency of AD patients to misattribute imagined actions to enacted ones was reported in another study, in which patients had to decide whether they previously placed or imagined placing objects in a bag [19]. Similar results were reported by O'Connor et al. [22] who invited AD patients to listen to action statements (e.g., “fold this paper”) and to engage in one of three activities: listen to the statements being read, perform the actions, or imagine performing the actions. On a second session, participants were invited to imagine action statements from the first session, as well as new action statements. At a recognition test, participants were invited to determine whether action statements were or were not performed during the first session. Results demonstrated that imagining performing actions during the second session increased the tendency of AD patients to falsely remember actions as having been performed. O'Connor et al. [22] attributed these results to the imagination inflation effect, i.e., imagining an event which never happened can increase tendency to falsely remember that it actually occurred [23].

The evidence reviewed here indicates not only false item memory in AD but also false context memory. False context memory in AD can be observed even when patients try to remember whether information was previously encountered or imagined. Below, we discuss how false memory can even concern memories for long-term events.

### 2.3. False Autobiographical Memory

False memory in AD may also involve misattributions of entire autobiographical events, as suggested by research on confabulations. Consensus regarding the causes of confabulations or even their classification and definition does not appear to have been reached, as proposed by Nahum, Bouzerda-Wahlen, Guggisberg, Ptak, and Schnider [24] who explored the dissociations between and the cognitive correlates and mechanisms of the proposed different forms of confabulation. Confabulations can be however generally defined as the emergence of memories of events and experiences that in reality never took place [25]. Confabulations in AD have been typically evaluated with the confabulation battery [26], consisting of questions concerning general personal knowledge (e.g., patient's name, date, and place of birth) and questions concerning specific autobiographical events (e.g., “can you remember that family event”?). Using this battery, we and others reported confabulations in AD patients [27–31]. According to one study [31], patients with severe confabulations falsely create events extended to hours or days; for instance, a patient may claim that she spent the weekend in a friend's house even though she was hospitalized during the weekend.

A distinction has been proposed between provoked and spontaneous confabulations [32, 33]. Provoked confabulations refer to memory fabrications often provided in response to requested information, whereas spontaneous confabulation is unprovoked. While most research has focused on provoked confabulations, one study has evaluated spontaneous confabulations [29]. This study asked nursing and medical staff to rate these confabulations, with results demonstrating only occasional appearance of spontaneous confabulations in AD participants, which, interestingly, were significantly correlated with temporal orientation. This correlation can be interpreted in light of the temporal consciousness model [34], according to which confabulators suffer from a disturbed sense of chronology, so that they can retrieve the content of events but not their order of occurrence. As a consequence, they misattribute features of an event that occurred at one time to another event that occurred at another time. The relationship between confabulations and temporal orientation in AD, as observed in the study of El Haj and Larøi [29], also mirrors the orbitofrontal reality filtering hypothesis [35, 36]. This hypothesis emphasizes the temporal difficulties of confabulating patients who fail to place themselves correctly in time and space and, therefore, are confused about reality. Supporting this hypothesis, research has demonstrated that spontaneous confabulation and disorientation in nondemented amnesics mostly share a common mechanism, i.e., defective orbitofrontal reality filtering [24].

In summary, research on confabulations in AD has demonstrated how false memories can affect long-term personal memories (i.e., autobiographical memories). Till now, we demonstrated how false memory in AD can affect item memory, context memory, and autobiographical memory. Below, we discuss phenomenological characteristics of false memories in AD to demonstrate how they contribute to the occurrence of false item memory, false context memory, and false autobiographical memory in the disease.

## 3. Phenomenology of False Memory in AD

**Figure 1:**
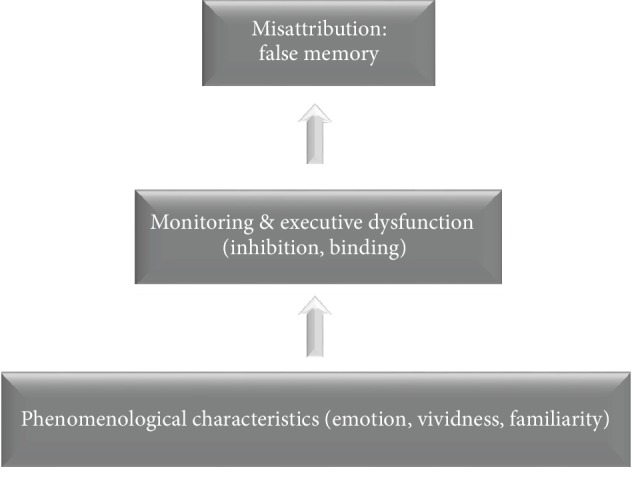
We propose that phenomenological characteristics of memories contribute to misattributions in Alzheimer's Disease (AD). More specifically, decline in inhibition in AD results in a difficulty to suppress irrelevant information during memory monitoring, especially when the irrelevant (i.e., false) information is characterized by high emotion, vividness, or familiarity. We also propose that binding deficits in AD decrease the ability to retrieve relevant contextual details, leading to memory monitoring errors and misattributions.

This review attempts to explain how phenomenological characteristics of false memories contribute to their misattribution, i.e., errors in which an event is misattributed to another context. As illustrated in Figure 1, this misattribution can be due to several phenomenological characteristics of memories, namely, the emotional load of a memory and its vividness and familiarity.

### 3.1. Emotional Load

To begin with emotion, memories of highly emotional events are vivid and lasting but not necessarily accurate, and, under some conditions, emotion increases susceptibility to false memories [37]. A study has demonstrated that it is possible to create false memories for emotional events such as witnessing a violent fight or being hospitalized [38]. This study compared memories for emotional events described by participants who actually experienced these events and memories of participants who were induced to believe they had experienced these events. Results demonstrated that true and false memories were nearly indistinguishable. Supporting these findings, research has demonstrated that emotional arousal can impair memory accuracy and increase susceptibility to misinformation [39].

The effect of emotional load on false memories may depend on how important the event is to the individual (i.e., its salience). According to Kaplan, Van Damme, Levine, and Loftus [40], emotions high in motivational intensity (e.g., fear, anger) powerfully direct attention to features of events that are of high importance, which predisposes to false memories concerning features of events that are peripheral to their motivational goals. This hypothesis fits with research demonstrating that, with high emotional arousal, people focus almost exclusively on features of events that are of central importance (e.g., weapon, survival), resulting in low memory for peripheral details [41, 42]. Interestingly, AD patients tend to prioritize retention of emotional memories [2, 43, 44]. For instance, when Sundstrøm [45] invited AD patients to try to remember neutral items and emotional items (i.e., gifts offered to the patients), results demonstrated high recall for the emotional items. In another study [46], AD patients were invited to rate neutral and emotional adjectives describing themselves; results demonstrated high recall for emotional adjectives. Another study demonstrated that AD patients rate their autobiographical memories as “highly emotional” [47]. These outcomes suggest relatively high retention for emotional information in AD. This preferential retention of emotional information may leave AD patients susceptible to false memories concerning features of events that are peripheral to the central information. An alternative hypothesis was proposed by Gallo, Foster, Wong, and Bennett [48] who suggested that AD spares processing of emotional events but limits the ability to retrieve their encoding context.

### 3.2. Vividness

The emotional load of memory can be associated with high vividness, i.e., high visual clarity and visual intensity of the memory. Vividness has been considered as a core phenomenological characteristic of autobiographical memory [49–52]. Memories for highly emotional events tend to remain vivid, even after delays of as long as 50 years [53], and vividness of memories has been linked to suggestibility to false memories [54, 55]. A study reported that use of imagery techniques increased the proportion of false childhood memories in college students [56]. In a similar vein, a study reported a relationship between scores on a self-report scale of vividness of mental imagery and false memories in young healthy participants [54]. In another study, young healthy participants were invited to retrieve pictures and words during low-stress and high-stress conditions; intrusions during recall were characterized as false memories [57]. Results demonstrated that participants with high vivid imagery were vulnerable to false memories, especially in the high-stress condition. These studies suggest an intimate relationship between vividness and false memories, such that the ability to create vivid images makes it more likely that people will believe that they have actually experienced the event. This account can be supported by the Source Monitoring Framework [3, 58] according to which increased vividness of images makes them less distinctive from percepts, which may lead to confusion between imagined and perceived events.

Regarding AD, vividness of some events, at least emotional ones, may result in difficulty in retaining the origin of information (e.g., whether they previously encountered that information in that context or not), resulting in false context memories. This assumption can be supported by research demonstrating high vividness of emotional memories in AD [47], as well as research demonstrating preservation of the ability to generate basic visual images in mild AD. For instance, Hussey, Smolinsky, Piryatinsky, Budson, and Ally [59] assessed visual imagery by asking patients with mild AD to generate a mental image of an object (e.g., a pen) presented as a word and determine whether the object is taller than it is wide. Results demonstrated no differences between AD patients and healthy elderly individuals on this task, suggesting a preservation of the ability to generate basic visual images. A similar conclusion was reached in a study, in which patients with mild AD were invited to imagine objects and decide whether these objects were bigger or smaller than a shoebox [60].

### 3.3. Familiarity

False memories in AD may be triggered by the familiarity of the retrieved event. This hypothesis stems from the dual process theories for recognition memory, according to which two distinct processes contribute to discriminate the origin of a memory: recollection and familiarity [61]. Recollection refers to retrieval of the context, in which a specific event was previously encountered, whereas familiarity refers to a contextual sense that an event has been previously encountered. The relationship between false memory and familiarity can be evaluated with the Remember/Know paradigm [62, 63], in which participants indicate whether during retrieval they reexperience previously encoded episodes with details (i.e., “Remember” response) or whether retrieval triggers a feeling of familiarity in the absence of any recollection (i.e., “Know” response). According to Tulving [63], “Remember” responses indicate a state of autonoetic consciousness in which the past is mentally relived. Using this paradigm, as well as the Deese–Roediger–McDermott paradigm, a study reported significant relationship between false memories and familiarity of the retained information in healthy adults [7].

Considering AD, false memories may be associated with familiarity-based judgements. This account can be supported by a study, in which reliving was assessed by asking patients to retrieve personal memories; after memory retrieval, AD patients were invited to provide a “Remember” response, if they believe that they retrieved the memories with their encoding context, or a “Know” response if they believe that the memory is simply familiar [31]. Results demonstrated a significant negative correlation between confabulations and “Remember” responses, i.e., high levels of confabulations were associated with a diminished ability to relive the past. These findings suggest a relationship between confabulations in AD patients and impairments in their ability to accurately relive the context, in which memories were encoded. According to Noel et al. [31], the diminished subjective experience of memories in AD leads to difficulties in associating them with their encoding context, predisposing to a sense of familiarity for irrelevant memories and, consequently, confabulations. This account can be supported by a body of research revealing decreased ability of AD patients to relive the past and increased sense of familiarity [43, 64–68]. Interestingly, research has demonstrated that AD patients may rely on familiarity during recognition [8] and that relying on the sense of familiarity increases false alarms in AD [16, 69, 70]. Taken together, false memories in AD patients can be associated with reliance on familiarity and a decreased ability to relive the context in which memories were encoded.

The evidence reviewed suggests how phenomenological characteristics of memories (e.g., emotion, vividness, and familiarity) may result in misattributions of item memory, context memory, and autobiographical memory in AD. We next provide a detailed description of these misattributions.

## 4. Monitoring of False Memory in AD

This review (see Figure 1) proposes that, when judging the origin of memories, AD patients rely on the phenomenological characteristics of these memories. More specifically, when retrieving events triggering high emotional load, high vividness, or high familiarity, AD patients encounter difficulties in retrieving the appropriate context of these events. This conclusion is based on the above-reviewed evidence demonstrating how emotional load, vividness, and familiarity influence memory monitoring in AD. Regarding memory monitoring, our hypothesis can be compared with the Source Monitoring Framework [3] which defines the “monitoring” process as a key mechanism involved in the attribution of the origin of memories. However, our hypothesis can be distinguished from the Source Monitoring Framework in that we provide insight into the involvement of phenomenological characteristics in false memory monitoring in AD. Another distinction is that we provide insight into specific executive functions that may underlie memory monitoring, inhibition, and binding.

To begin with inhibition, memory monitoring in AD has been found to be related with the ability to suppress irrelevant information. This relationship was revealed in a study assessing different levels of source monitoring in AD [19]. In that study, AD patients were invited to remember whether the experimenter had previously placed objects in a bag with a black or white gloved hand; in another task, they were invited to remember whether they had previously placed or imagined themselves placing objects in a bag. Results demonstrated a significant correlation between source monitoring and performance on the inhibitory Stroop task (1935). This suggests that the difficulty of AD patients to suppress irrelevant information during retrieval is associated with (or even result in) the retrieval of irrelevant information when monitoring the origin of memories [71]. A similar account was offered by [72]) who suggested that impairment in executive function, especially in inhibition, contributes to the inability of patients with AD to suppress false memories. An important challenge for memory monitoring is the competition between appropriate and inappropriate information, especially when such information is characterized by high emotional load, vividness, and familiarity. Due to a decline in inhibition, AD patients lack effective control mechanisms that may reduce this competition, resulting in failures to suppress irrelevant information during retrieval and consequently, in false memories. We propose that a diminished ability to suppress irrelevant information hampers retrieval of appropriate memory by activating false information at the expense of relevant ones, a hypothesis supported by a large body of research demonstrating decline of inhibition in AD [73–75], as well as research demonstrating difficulties of AD patients to suppress irrelevant information in memory [76–78].

Another executive function that may be involved in failed monitoring of false memory in AD is binding. Generally speaking, memory monitoring is thought to be dependent on a feature-binding process connecting contextual features to a central event [79]. The binding mechanisms that hold contextual information together are important when retaining contextual details associated with an event; binding also enables retrieving memories that contain varying levels of complexity, from simple memories of an item to complex autobiographical memories [80]. We propose that memory retention requires processing of different contextual information (e.g., where, when, and how an event was encoded), and binding these features together differentiates one event from another, providing memory specificity. Consequently, binding deficits, as observed in AD, make memories less specific and distinguishable, resulting in a compromised ability to monitor the origin of memories and leading to the appearance of false memories. In other words, binding deficits in AD decrease the ability to retrieve relevant contextual details, leading to source monitoring errors and facilitating false memories. This hypothesis can be supported by research demonstrating a significant decline of binding in AD [81, 82].

In summary, this review highlights the involvement of executive dysfunction in distortions of memory monitoring and, consequently, in occurrence of false memories in AD. More specifically, this review proposes that decline in inhibition in AD results in a difficulty to suppress irrelevant information during memory monitoring, especially when the irrelevant (i.e., false) information is characterized by high emotion, vividness, or familiarity. This review also proposes that binding deficits in AD decrease the ability to retrieve relevant contextual details, leading to distortions of memory monitoring and, consequently, false memories.

## 5. Discussion

This review provides a hypothesis for understanding cognitive processes involved in the occurrence of false memories in AD. We cover a whole range of false memories in AD (i.e., false item memory, false context memory, and false autobiographical memory), explain how phenomenological characteristics of information can influence memory misattributions, leading to false memory in AD, and offer a description of the cognitive mechanisms potentially underlying false memory in AD.

By describing how phenomenological characteristics may contribute to the occurrence of false memories in AD, this review paves the way for studies on characteristics that may prevent occurrence of false memory. For instance, because familiarity may result in misattributions, novelty and distinctiveness may result in correct attributions. A clinical trial could assess whether AD patients adopting a distinctiveness heuristic process based on specific features of the encoding context (e.g., this caregiver is new because she/he is wearing the training uniform) have fewer false memories. An intervention invoking a distinctiveness heuristic process may improve memory monitoring in AD, as suggested by research demonstrating that AD patients may use distinctiveness heuristics to reduce false memories [83], at least when information is not closely semantically related [84]. Whereas this line of research has been concerned with false item memory and false context memory, little is known about the effects of distinctiveness heuristic on false autobiographical memory in the disease. Research on the effects of metacognitive strategies may also build upon a study by Deason et al. [85] who invited patients with mild cognitive impairment to simulate going through a grocery store on two conditions. On the first condition, patients were invited to decide whether they needed to buy items. On the second condition, patients were invited to decide whether they needed to buy items after a series of questions guiding them through a recall-to-reject strategy (e.g., “is this item familiar to you?”). Results showed that the metacognitive instructions significantly reduced false memories. Because these results were reported for patients with mild cognitive impairment, research may consider replicating this study in AD.

Another feature potentially associated with false memories is motivation. To understand the occurrence of false memories, it is essential to consider the goals and motivations associated with these memories and the relevance of the information being falsely remembered to the patients' goals and beliefs. Patients may experience false memories related to the pursuit of a goal or to justify their beliefs and plans. For instance, a patient may falsely remember that she spent the last weekend with her family as she used to do, instead of the hospital, because she may have preferred the former over the latter. Affective factors should also be considered, such as the patients' need to reconnect with loved ones; for instance, a patient may falsely remember that her (deceased) parents visited her due to her emotional need to see them. Although these factors are important to be considered by clinicians dealing with false memory, empirical research is needed to examine how these motivational factors may contribute to false memory in AD, especially research on how false memories can be created to support patients' goals, beliefs, and needs. Such research may build upon previous theoretical models proposing that memory retrieval is dependent on goals and beliefs. For instance, the self-memory system [4] postulates that memory retrieval depends on two processes: coherence and correspondence. Coherence implies that the retrieved memory should be coherent with goals and beliefs, whereas correspondence implies that the retrieved memory should correspond to a previously experienced event, i.e., to the accurate encoding conditions of the event. These two processes may explain how patients produce false memories to make sense of their reality. In our view, AD patients may produce false memories based on previous experiences (i.e., correspondence) as an attempt to justify their goals and beliefs (i.e., coherence), especially when the environment does not readily provide them with a sufficient explanation of an event. For instance, when getting lost in a unit of a retirement home, a patient may attempt to explain why she is found there by falsely remembering living in that unit (i.e., correspondence). This suggestion is particularly relevant to false autobiographical memory, as opposed to false item memory or false context memory.

Regardless of its clinical implications, this review can be enriched by evaluating other phenomenological characteristics that may contribute to false memories in AD. We suggest that belief-worthiness may also play a role in the occurrence of false memories in AD. Although this hypothesis can be supported by research demonstrating deficits of awareness in AD patients (i.e., anosognosia) [86–89], this review did not include belief-worthiness in the phenomenological characteristics owing to scarce research on belief-worthiness and false memory in AD, an issue that should be addressed by future research. Another issue that should be addressed is assessment of executive functions, other than inhibition and binding, which may be involved in false memory monitoring. One potential candidate is updating, as this function has been considered as a core executive function [90]. It is likely that AD patients produce false memories as a failure to update current knowledge; for instance, a patient may falsely remember that she was at work on the previous day, whereas, in reality, she is retired. This false memory may be due to failure to update her semantic autobiographic memory with new events (i.e., that the patient is currently retired). This updating failure can be intimately linked with anterograde amnesia, i.e., the inability to form new memories, that characterizes AD [91–93]. Because this updating failure has been linked with difficulties of AD patients to construct new roles and beliefs [94], it would be of interest to investigate whether false memories mirror old rather than new goals and beliefs.

This review highlights multiple types of false memories (i.e., false item memory, false context memory, and false autobiographical memory) and multiple variables contributing to these false memories (e.g., emotion, vividness, and familiarity). These variables may contribute to false memories by simply leading to a faint memory trace, in agreement with the Fuzzy Trace Theory [15, 95]. According to this theory, there are two types of memory traces, verbatim and gist. While verbatim traces are typically vivid and include realistic representations of the encoded information that support accurate recall, gist traces capture general meaning of the to-be-remembered information. Decline in the ability to produce verbatim traces leads to faint memory traces and, consequently, false memories. In other words, decline in the processes proposed by our review (e.g., vividness) may simply lead to a faint memory trace and, consequently, to false recognition. Also, false memories in AD can simply be the consequences of the general cognitive decline in AD, in particular, as proposed by our review, the decline of monitoring and executive function.

## 6. Conclusion

At the cognitive level, AD has been mainly considered as a memory disorder [93], and, not surprisingly, there is a large body of research devoted to the study of memory distortions in the disease. Accordingly, false memory has received particular attention owing to its consequences for the patients' daily life. To provide a comprehensive account of the current knowledge on false memory in AD, we constructed our review as an attempt to synthesize this knowledge into a framework allowing better understanding of the different categories of false memories and of the cognitive mechanisms that may generate these memories in AD. This framework refers to the mild to moderate stages of AD, as the evaluation of memory in the advanced stages of AD is challenging. In particular, the emergence of cognitive decline in other cognitive domains (i.e., the appearance of aphasia, inattention, and executive dysfunction) significantly hampers the patients' ability to perform on memory assessment tasks. However, this framework paves the way for research on potential benefits of false memory in AD, i.e., how patients may produce false memories to support their current goals and beliefs. In our view, this line of research may inform and modify the clinical management of false memory in AD, minimize associated suffering, and hopefully maximize potential benefits.
